# Suppressed microRNA-96 inhibits iNOS expression and dopaminergic neuron apoptosis through inactivating the MAPK signaling pathway by targeting CACNG5 in mice with Parkinson’s disease

**DOI:** 10.1186/s10020-018-0059-9

**Published:** 2018-11-28

**Authors:** Yue Dong, Li-Li Han, Zhong-Xin Xu

**Affiliations:** 1 Department of Neurology , China-Japan Union Hospital, Jilin University, No. 126, Xiantai Street, Erdao District, Changchun, 130012 Jilin Province People’s Republic of China; 2 Department of Neurology, Cangzhou Central Hospital, Cangzhou, 061000 People’s Republic of China

**Keywords:** MicroRNA-96, CACNG5, Parkinson’s disease, MAPK signaling pathway, Inducible nitric oxide synthase, Dopaminergic neuron

## Abstract

**Background:**

There have been a number of reports implicating the association of microRNAs (miRs) and the MAPK signaling pathway with the dopaminergic neuron, which is involved in the development of Parkinson’s disease (PD). The present study was conducted with aims of exploring the role of miR-96 in the activation of iNOS and apoptosis of dopaminergic neuron through the MAPK signaling pathway in mice with PD.

**Methods:**

The miR and the differentially expressed gene in PD were screened out and the relationship between them was verified. A mouse model of PD induced by MPTP and was then constructed and treated with miR-96 mimic/inhibitor and CACNG5 overexpression plasmid to extract nigral dopaminergic neuron for the purpose of detecting the effect of miR-96 on PD. The TH and iNOS positive neuronal cells, the apoptotic neuronal cells by TUNEL staining, and expression of miR-96, CACNG5, iNOS, p38MAPK, p-p38MAPK, c-Fos, Bax, and Bcl-2 in substantia nigra dopaminergic neuronal tissues were evaluated.

**Results:**

The results obtained from the aforementioned procedure were then verified by cell culture of the SH-SY5Y cells, followed by treatment with miR-96 mimic/inhibitor, CACNG5 overexpression plasmid and the inhibitor of the MAPK signaling pathway. CACNG5 was confirmed as a target gene of miR-96. The inhibition of miR-96 resulted in a substantial increase in nigral cells, TH positive cells and expression of CACNG5 and Bcl-2 in nigral dopaminergic neuronal tissues, and a decrease in iNOS positive cells, apoptotic neuronal cells, and expression of iNOS, p38MAPK, p-p38MAPK, c-Fos, and Bax.

**Conclusion:**

The above results implicated that the downregulation of miR-96 inhibits the activation of iNOS and apoptosis of dopaminergic neuron through the blockade of the MAPK signaling pathway by promoting CACNG5 in mice with PD.

## Background

Parkinson’s disease (PD) is a major neurodegenerative movement disorder affecting over 1% old people over 60 years old (Crabtree and Zhang 2012). It is the second most commonly occurring type of neurodegenerative disease following Alzheimer’s disease with a lifetime risk of 4–5% (Mullin and Schapira 2013). The pathogenesis of PD is complex and age-related, which involves both genetic and environmental factors (Fujita et al. 2014). PD is characterized by a variety of movement related symptoms including bradykinesia, resting tremor, stiffness of movement and postural instability (Lewis and Cookson 2012). The most effective treatment for PD is Levodopa, which is effective in controlling the motor symptoms of PD, while dopamine replacement therapy is beneficial in treating motor symptoms associated with the early stages of the disease (Fuentes et al. 2009; Muller 2013). Midbrain dopaminergic neurons play an essential regulatory role in several brain functions, including emotion, cognition and voluntary movements (Villaescusa et al. 2016). The progressive loss of midbrain dopaminergic neurons in the substantia nigra results in a decrease in the levels of dopamine in the striatum, contributing to the dysfunction in the motor system that is observed in PD (Rhee et al. 2011). Recently, microRNAs (miRNAs) have been found to play a key role in the function, and development of the nervous system as well as the pathogenesis of several diseases of the nervous system, and therefore they have been used as a therapeutic agent in PD (Harraz et al. 2011).

MicroRNA-96 (MiR-96) is a member of the miR-183 family which maps to chromosomal region 17 and functions as an oncogene, facilitating the progression of malignant tumors (Li et al. 2015; Rapti et al. 2016). Zhang et al. demonstrated that miR-96 promotes cell proliferation, invasion and migration in breast cancer by targeting reversion-inducing cysteine-rich protein with Kazal motifs (Zhang et al. 2014). MiR-96 is an essential gene regulatory network element of the auditory system which is required for the functional maturation of the peripheral and central auditory system (Schluter et al. 2018). miR-183/96 is indispensable for photoreceptor maturation and maintenance through modulation of fine-tuning of Slc6a6 expression (Xiang et al. 2017). The overexpression of miR-96 has been reported to provide a great deal of protection from brain damage in status epilepticus through the inhibition of Atg7 and Atg16L1 expression and autophagosome formation in the hippocampus (Gan et al. 2017). Calcium voltage-gated channel auxiliary subunit gamma 5 (CACNG5) is a member of γ subunit in voltage-gated L-type calcium channels and multiple nucleotide mutations in the CACNG5 gene have been reported in patients with schizophrenia, suggesting a potential association between the CACNG5 gene and schizophrenia; however, the underlying mechanism is yet to be determined (Guan et al. 2016). There’s evidence demonstrating that CACNG5 is expressed in brain tissues, and the mutation of CACNG5 is associated with schizophrenia (Guan et al. 2016), and CACNG5 is also involved in the development of spinal cord ependymoma by regulating MAPK signaling pathway (Wu et al. 2018). As crucial enzymes at the intersection of several biological pathways, mitogen-activated protein kinases (MAPK) can regulate cancer cell proliferation, differentiation and survival (Huang et al. 2008). The activity of an extracellular stimuli-responsive kinase, c-Jun NH2-terminal kinase and p38, the three members of the MAPK family, has been shown in blood platelets (Flevaris et al. 2009). Inducible nitric oxide synthase (iNOS) produces large quantities of nitric oxide and is induced in microglia and macrophages in response to inflammatory mediators (Sierra et al. 2014). iNOS is regulated by MAPK and the overexpression of MAPK could potentially result in the enhancement of gene expression of iNOS in the glial cell (Vause and Durham 2009). The present study was conducted with aims of exploring whether miR-96 affects the development of PD. We hypothesized that downregulation of miR-96 exerts an inhibitory effect on iNOS activity and dopaminergic neuron apoptosis during PD, and the underlying mechanism may relate to the CACNG5-mediated MAPK signaling pathway.

## Materials and methods

### Ethics statement

The present study was performed in strict accordance with the ethics committee of China-Japan Union Hospital, Jilin University. All experiments were conducted with maximum effort in minimizing animal death and suffering.

### Behavioral observation of each mouse

**Fig. 1:**
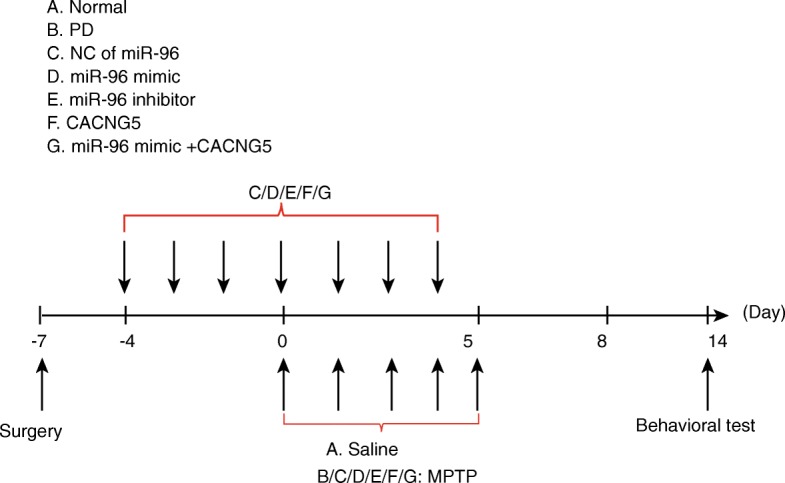
The flowchart of model establishment and behavioral test. The horizontal axis represents the time points after the operation

A total of 70 healthy, clean male C57BL/6 mice (weight: 20 g to 25 g; age: 8–10 weeks) were provided by the experimental animal center of the Bethune Medical Department (Jilin, China) and kept at (20 ± 2)°C (light for 14 h and darkness for 10 h). There were no abnormal findings in the mice following a one week period of adaptive feeding. Following fasting for 12 h, the mice were anesthetized through the intraperitoneal injection of 3% pentobarbital sodium. Next, the skull of the mice was fully exposed in accordance with the book written by GEORGE et al. (Ayuk et al. 2016), after which a triangle-edged needle was used to drill through the skull in the lateral ventricle area of the skull surface. Subsequently, the right lateral ventricle of mice was covered by an appropriate stainless-steel casing tube and the stylet was used to avoid blocking. The casing tube was fixed by glue and dental base acrylic resin powder. During the 3 d postoperative recovery period, 20 thousand units of penicillin was intraperitoneally administered to avoid infection. Afterwards, the 70 mice were randomly assigned into seven groups (10 mice in each group) and the following drugs were administered: normal group (mice without operation, injected with normal saline), PD (mice injected with 1-methyl-4-phenyl-1, 2, 3, 6-tetrahydropyridine (MPTP, Sigma-Aldrich, St. Louis, MO, USA)) group, negative control (NC) group (PD mice injected with MPTP and miR-96 scramble NC), miR-96 mimic group (PD mice injected with MPTP and miR-96 mimic sequence to overexpress the miR-96 level), miR-96 inhibitor group (PD mice injected with MPTP and miR-96 inhibitor sequence to inhibit the miR-96 level), CACNG5 group (PD mice injected with MPTP and CACNG5 overexpression virus, NBP2–05372, Shanghai yaoyun biological technology Co., Ltd., Shanghai, China), and miR-96 mimic + CACNG5 group (PD mice injected with MPTP, miR-96 mimic sequence and CACNG5 overexpression virus). The mice received their corresponding injections for 8 d. The sequences of miR-96 mimic and inhibitor were RNA sequences (miR-96 mimic: CAAUCAUGUGCAGUGCCAAUAU, miR-96 inhibitor: ATATTGGCACTGCACATGATTG), and the CACNG5 sequence was DNA sequence. All of the transfected sequences were purchased from Shanghai Sango Biotech Co., Ltd. (Shanghai, China). The pole climbing ability of each mouse was evaluated by conducting a pole test. For a brief description of this process, the mice were placed in an 80-cm pole and three specific times were recorded, including: (1) the time required for the mice to climb up the upper part of the pole; (2) the time required for mice to climb up the lower portion of the pole; (3) the time required for mice to climb up the entire pole. Their behaviors were also assessed according to the following standards: 3 points for completing an action within 3 s; 2 points within 6 s and 1 point beyond 6 s. The final point was expressed as the sum of three points. This experiment was conducted three times. The mice were euthanized on the 14th day following MTPT injection. The experiment procedures including model establishment, animal treatment, and behavioral test were thoroughly illustrated in the Fig. 1.

### Hematoxylin-eosin (HE) staining

Following the successful establishment of the models, the mice were anesthetized through the intraperitoneal injection of 3% pentobarbital sodium. After anesthetic maintenance, the mice were rapidly decapitated, the cranial cavity of mice was opened and the brain was removed. The substantia nigra dopaminergic neuronal tissues were then isolated from the brain on an ice plate, washed by normal saline, after which the water was removed with the use of absorbent papers. Substantia nigra dopaminergic neuronal tissue blocks (1 mm) were then fixed in 4% paraformaldehyde overnight and dehydrated, paraffin-embedded, and sliced into serial sections (6 μM). Afterwards, the substantia nigra dopaminergic neuronal tissue sections were stained by HE (YB-8499, YUBO Biological Co., Ltd., Shanghai, China) through the following procedure: sections were dewaxed in xylene twice (15 min each time), in absolute ethanol twice (5 min each time), in 90% ethanol for 5 min, 80% ethanol for 5 min, and then washed for 5 min; hematoxylin stain was then carried out for 5 min, followed by washing by water until a blue color was obtained in the sections. Subsequently, the sections were rapidly differentiated in 1% hydrochloric acid-ethanol and then washed by water till a blue color was observed in the sections. Then, the sections were immersed in 80% ethanol for 5 min, 90% ethanol for 5 min, and absolute ethanol twice (5 min each time) for dehydration followed by xylene clearance twice, each session lasting 15 min. Finally, the sections were sealed with neutral balsam and the pathological changes in the HE stained sections were observed and photographed in the seven groups under an HE optical microscope. JD-801 morphological image analysis system (JEDA Science and Technology Development Co., Ltd., Nanjing, Jiangsu, China) was used to collect the sections in each group (100 ×), after which they were photographed randomly. The above experiment was conducted three times.

### Immunohistochemistry

The samples that had been fixed by 10% formaldehyde were embedded with paraffin, and sliced into serial sections (4 μm). Substantia nigra dopaminergic neuronal sections were baked in a 60 °C oven for 1 h, dewaxed in conventional xylene and then dehydrated in gradient alcohol. After being washed by phosphate buffer saline (PBS), the sections were incubated in 3% H_2_O_2_ (Sigma-Aldrich, St. Louis, MO, USA) at 37 °C for 30 min, and then boiled in 0.01 M citrate buffer solution at 95 °C for 20 min. Once the sections cooled down to room temperature, they were washed by PBS. The sections were then sealed with normal goat serum solution at 37 °C for 10 min, added with rabbit polyclonal antibody iNOS (1: 100, ab15323, Abcam, Cambridge, UK) and tyrosine hydroxylase (TH) (1: 2000, ab129991, Abcam, Cambridge, UK), reacted at 4 °C for 12 h, and incubated at 4 °C overnight. Following that, the sections were washed by 0.2 mol/L of PBS (pH 7.4) three times (5 min each time) and incubated in horseradish peroxidase labeled second antibody goat anti-rabbit (DF7852, Shanghai yaoyun biological technology Co., Ltd., Shanghai, China) for 30 min after being dried. The chromogenic reagent was prepared as follows: A, B, and C reagent of diaminobenzidine (DAB, DA1010, Beijing Solarbio Science & Technology Co., Ltd., Beijing, China) were dropped into 1 mL distilled water respectively and stored in a 4 °C refrigerator after being mixed. The sections were then washed by 0.2 mol/L of PBS (pH 7.4) three times (5 min each time) and developed by DAB in a dark room for 8 min and washed by running water. Next, the sections were stained with hematoxylin, dehydrated, cleared, sealed and observed under an optical microscope, after which three different fields of visions (200 ×) with the same size in each section were selected in order to calculate the positive cells with the use of the image analysis software (Nikon Corp., Tokyo, Japan). Immunohistochemistry was also employed for the detection of the TH and iNOS co-localization. After being fixed in paraformaldehyde, the tissues were paraffin-embedded, sectioned and dewaxed, and washed with phosphate buffered saline (PBS) 3 times, each being conducted for 5 min. The cooling section was then treated with normal goat serum (10% PBS and 0.3% Triton X-100) for 10 min. Then, the section was incubated with the primary antibody diluted with PBS containing 0.3% Triton X-100 and 1% normal goat serum overnight. The section was then washed with PBS 3 times, 5 min each and incubated at room temperature, after which the sections were treated with the secondary antibody carbocyanine 3-labeled IgG. The polyclonal antibody of TH and iNOS was used as the primary antibody detected by the monoclonal antibody. The section was washed and mounted on coverslips, which was then analyzed with the use of a confocal laser scanning microscope (LSM 700, Carl Zeiss, Jena, Germany).

### Terminal deoxynucleotidyl transferase-mediated dUTP nick end labeling (TUNEL) staining

The sections in each group were then sealed in 3% H_2_O_2_ at room temperature for 15 min following dewaxing by water. Afterwards, the sections were digested with protease K at 37 °C for 40 min followed by PBS washing and a reaction in a terminal deoxynucleotidyl transferase solution for 1 h. The conversion solution was added and the reaction continued for 30 min. Following PBS washing, the sections were developed by DAB for 3 min until satisfactory staining results were obtained. The apoptotic cells appeared to have a black or blue-black color following TUNEL staining (KGA7022, Nanjing KeyGen Biotech Co. Ltd., Nanjing, Jiangsu, China). Five non-continual nigral dopaminergic neuronal tissues with equivalent interval were analyzed from each mouse, after which five high power fields of visions (400 ×) were selected in order to observe the expression of positive cells in nigral dopaminergic neuronal tissues following TUNEL staining.

### Dual luciferase reporter gene assay

The biological prediction website http://www.targetscan.org was used for the analysis of the target gene of miR-96. HEK-293 T cells (AT-1592, American Type Culture Collection (ATCC, Manassas, VA, USA) were inoculated in a 24-well plate and cultured for 24 h. The total RNA was extracted and reversely transcribed into cDNA. Polymerase chain reaction (PCR) amplification was carried out to obtain the full-length sequence of CACNG5 3’-UTR with cDNA used as the template. The primer was synthesized in accordance with the sequences of CACNG5 and the amplification was conducted, with genomes extracted from HEK-293 T cells used as template. After amplification, the product was cloned to the downstream of pmiRRB vector luciferase encoding genes using the endonuclease. Wild-type (wt)-CACNG5–3’UTR (AGCCCCGCCUCUAGAGUGUUAAU) and Mutant (mut)-CACNG5–3’UTR (AGCCCCGCCUCUAGTCAUTGUAU) were obtained and co-transfected into HEK-293 T cells with miR-96 mimic and NC respectively. After a 48-h transfection, the medium was removed and the cells were washed by PBS twice. Subsequently, cell lysis was conducted and luciferase activity was evaluated using the Dual-Luciferase® Reporter Assay System (E1910, Promega Corp., Madison, WI, USA). The firefly luciferase activity and renilla luciferase activity were assessed by adding a total of 50 μL firefly luciferase solution and 50 μL renilla luciferase solution into each 10 μL cell sample respectively. The relative luciferase activity was expressed as the ratio of firefly luciferase activity to renilla luciferase activity. This experiment was conducted three times.

### Cell culture and transfection

The SH-SY5Y cells were cultured in DMEM containing 10% new-born calf serum, and incubated in 5% CO_2_ at 37 °C. Prior to transfection, the cells at logarithmic growth phase received trypsin treatment and were inoculated in a 6-well plate at 1: 2.5 passage ratio and cultured with the antibiotic-free medium. Cell confluence reached about 80% the following day, during which time cell culture was carried out. CACNG5, miR-96 mimic and miR-96 inhibitor were transfected into the SH-SY5Y cells by liposome (Lipofectamine 2000) according to the instructions on the kit. The cells were then treated with a MAPK signaling pathway inhibitor, SB203580 (Selleck Company, Shanghai, China) and a NC group was set. After 24-h transfection, the expression levels of the corresponding proteins were detected.

### Reverse transcription quantitative polymerase chain reaction (RT-qPCR)

The total RNA in substantia nigra dopaminergic neuronal tissue samples was extracted using an RNA kit (10296010, Invitrogen, Shanghai, China) and the RNA was reversely transcribed into cDNA using a PrimeScript RT kit (RR014A, Takara Biomedical Technology Co., Ltd., Beijing, China). The reaction conditions of reverse transcription system (10 μL) were as follows: reverse transcription reaction was set at 42 °C for 30–50 min and reverse transcriptase inactivation reaction was set at 85 °C for 5 s. Primer sequences of miR-96, CACNG5, p38 mitogen-activated protein kinases (p38MAPK), c-Fos, iNOS, Bcl-2 associated protein X (Bax), Bcl-2, U6, and glyceraldehyde-3-phosphate dehydrogenase (GAPDH) were synthesized by Takara Biotechnology Ltd., (Dalian, Liaoning, China) (Table 1). The RT-qPCR reaction was conducted according to the instructions on the kit (KR011A1, TIANGEN Biotechnology Co. Ltd., Beijing, China). The reaction conditions were as follows: pre-denaturation at 95 °C for 5 min, a total of 30 cycles of at 95 °C for 40 s, at 57 °C for 40 s, at 72 °C for 40 s, and extension at 72 °C for 10 min and at 4 °C for 5 min. The reaction system consisted of 10 μL of SYBR Premix Ex Taq™ II, 0.4 μL of PCR Forward Primer (10 μM), 0.4 μL of PCR Reverse Primer (10 μM), 2 μL of DNA template and 7.2 μL of sterile distilled water. U6 was used as an internal reference of miR-96 relative expression, while GAPDH was considered as the internal reference of CACNG5, p38MAPK, c-Fos, iNOS, Bax, and Bcl-2 relative expression. The reliability of PCR results was evaluated using the melting curve analysis and threshold cycle (Ct) values were set. The 2^-ΔΔCt^ method was adopted to calculate the relative expressions of target genes with the following formula (Ayuk et al. 2016): ΔΔCt = [Ct (target gene) – Ct (reference gene)] _experiment group_ - [Ct (target gene) – Ct (reference gene)] _control group_.

**Table 1:** Primer sequences of RT-qPCR

Gene	Primer sequence (5’ - 3’)
miR-96	Forward: CGGCGGTTTGGCAATGGTAGAACT
	Reverse: CCAGTGCAGGGTCCGAGGTAT
CACNG5	Forward: GCGATTAGAGCCCAGCATGA
	Reverse: TGTCTTCAGGGGAGCACTTTC
p38MAPK	Forward: TCGAGACCGTTTCAGTCCAT
	Reverse: CCACGGACCAAATATCCACT
c-Fos	Forward: GGCTCTCCTGTCAACACACA
	Reverse: CCGCTTGGAGTGTATCTGTC
iNOS	Forward: ATGGACCAGTATAAGGCAAGC
	Reverse: GCTCTGGATGAGCCTATATTG
Bax	Forward: TGGAGCTGCAGAGGATGATTG
	Reverse: GCTGCCACTCGGAAAAAGAC
Bcl-2	Forward: TGCACCTGAGCGCCTTCAC
	Reverse: TAGCTGATTCGACCATTTGCCTGA
U6	Forward: GTGCTCGCTTCGGCAGCACAT
	Reverse: ATATGGAACGCTTCACGAAT
GAPDH	Forward: CCCATCACCATCTTCCAGGAG
	Reverse: CTTCTCCATGGTGGTGAAGACG

Note: *RT-qPCR* Reverse transcription quantitative polymerase chain reaction, *miR-96* microRNA-96, *CACNG5* Calcium voltage-gated channel auxiliary subunit gamma 5, *p38MAPK* p38 mitogen-activated protein kinase, *iNOS* Inducible nitric oxide synthase, *Bcl-2* B-cell lymphoma-2, *Bax* Bcl-2 associated protein X, *GAPDH* Glyceraldehyde-3-phosphate dehydrogenase

### Western blot analysis

Substantia nigra dopaminergic neuronal tissues (100 mg) that had been stored in a − 80 °C refrigerator from each group were placed in different glass grinders and ground into homogenate on an ice bath in 500 μL of tissue lysate (C1051, Guangzhou Weijia Science & Technology Co., Ltd., Guangdong, Guangzhou, China). Next, tissue samples were added with protein lysate, lysed at 4 °C for 30 min and gently shaken every 10 min. The tissue samples were then centrifuged at 4 °C for 20 min (12000 r/min), after which the lipid layer was removed and the supernatant was collected for subsequent use. The assessment of the total protein content was conducted in accordance with the bicinchoninic acid kit instructions (23250, Thermo Fisher Scientific Inc., Waltham, MA, USA), sub-packed and stored in a − 80 °C refrigerator. Once the protein denaturant was added, a total of 50 μg protein was collected from each group respectively and boiled for 10 min. Subsequently, protein samples underwent sodium dodecyl sulfate-polyacrylamide gel electrophoresis (SDS-PAGE) and were transferred from SDS-PAGE gel to a nitrocellulose membrane. The nitrocellulose membrane was sealed overnight in phosphate-buffered saline with Tween-20 (PBST) containing 10% skimmed milk powder and rinsed by PBST three times (5 min each time). The membrane was then incubated at 37 °C for 2 h, followed by the addition of the respective primary antibodies: rabbit polyclonal antibody CACNG5 (1: 100, ab135586), rabbit polyclonal antibody p38MAPK (1: 1000, ab170099), rabbit monoclonal antibody p-p38MAPK (1: 500, ab47363), rabbit polyclonal antibody c-Fos (1: 1000, ab134122), rabbit polyclonal antibody iNOS (1: 500, ab15323), rabbit monoclonal antibody Bax (1: 1000, ab32503), rabbit monoclonal antibody Bcl-2 (1: 1000, ab32124) and rabbit polyclonal antibody GAPDH (1: 2500, ab9485). All of the above primary antibodies were purchased from Abcam (Cambridge, MA, UK). After that, the membrane was incubated at 37 °C for 2 h, fully rinsed in PBST three times (10 min each time) and was further incubated at 37 °C for 2 h in horseradish peroxidase labeled goat anti-rabbit antibody immunoglobulin G (1: 1000, DF109489, Shanghai yaoyun biological technology Co., Ltd., Shanghai, China). Subsequently, the membrane was fully rinsed in PBST three times (10 min each time) and developed with the use of enhanced chemiluminescence (36208ES60, Amersham Biosciences, Piscataway, NJ, USA). The semi-quantitative analysis of grey levels was carried out by ImageJ software, with GAPDH used as an internal reference. The ratio of the grey level for target bands to control bands was used to determine the relative protein expression. The above procedure was conducted three times in each sample.

### Statistical analysis

All data processing was carried out with the use of the SPSS 21.0 software (IBM Corp. Armonk, NY, USA). Measurement data were shown as the mean ± standard deviation. Comparisons between two groups were performed by *t* test. Comparisons among multiple groups were conducted by one-way analysis of variance. Count data were analyzed by the chi-square test. *P* < 0.05 was considered as a statistically significant value.

## Results

### CACNG5 was confirmed as a target gene of miR-96 and a potential regulator of the MAPK signaling pathway

**Fig. 2:**
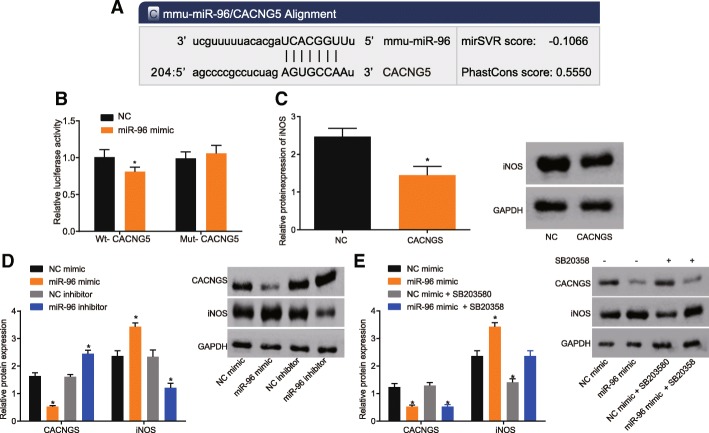
miR-96 is predicted to regulate the MAPK signaling pathway via CACNG5. **a** prediction of target relationship between miR-96 and CACNG5 promoter region; **b** the effect of miR-96 on WT-CACNG5 and Mut-CACNG5 luciferase reporter plasmid; **c** the effect of CACNG5 on the expression of iNOS protein; **d** and **e**, effect of miR-96 on the expression of iNOS protein. ^*^, *P* < 0.05 compared with the NC group. The test was repeated three times independently, and the non-paired t-test was used for comparison between the two groups; the one-way ANOVA analysis was used for comparison among multiple groups. miR-96, microRNA-96; CACNG5, calcium voltage-gated channel auxiliary subunit gamma 5; Wt, wild-type; Mut, mutant; NC, negative control

Based on the results from an online prediction website used to detect the relationship among miR-96, CACNG5 and the MAPK signaling pathway, a binding site was found to exist between miR-96 and CACNG5, as a result of which CACNG5 was identified as a target gene of miR-96. Following luciferase activity detection, the results showed that in cells carrying wt-CACNG5 plasmid, the luciferase activity of cells in the miR-96 mimic group was significantly decreased as compared with cells in the NC group (*P* < 0.05) (Fig. 2a). In cells carrying mut-CACNG5 plasmid, there was an insignificant change in the luciferase activity of cells between the miR-96 mimic group and the NC group (*P* > 0.05) (Fig. 2b). The levels of CACNG5 were elevated so that the expression of iNOS, the downstream molecule of MAPK was inhibited and this was done for the purpose of demonstrating that CACNG5 mediates the regulation of MAPK signaling pathway through miR-96 (Fig. 2c). We also found that overexpression of miR-96 resulted in the inhibition of CACNG5 expression and an increase in the protein level of iNOS. Inhibition of miR-96 promoted CACNG5 expression and decreased the protein level of iNOS (Fig. 2d). The MAPK signaling pathway was suppressed using SB203580 in order to demonstrate that CACNG5 regulates iNOS via the MAPK signaling pathway, and the results suggested that overexpression of miR-96 could down-regulate the level of CACNG5; however, there was no evident difference observed in the level of iNOS compared with the control group (Fig. 2e). These results suggested that miR-96 regulated iNOS expression by modulating CACNG5 via the MAPK signaling pathway.

### Inhibition of miR-96 ameliorates the symptoms of PD mice

Behavioral tests were conducted on the mice in each experimental group, in order to investigate the effects of miR-96 and CANG5 on MPTP-mediated PD mice. The results showed that the mice in the normal group were able to return to their normal position quickly after being turned and climb down the bottom of pole with good body coordination ability and presented with no abnormal behaviors during this experiment. However, the mice in the remaining six groups manifested certain symptoms similar to PD including staggering, quivering, pilo-erection, tail-erecting, foreleg elevation, hind leg opening, reduced activity and responses, and tardy movement. There was poor coordination ability and behaviors observed such as sliding, holding pole and remaining in this position for a while. The above symptoms were temporary and the mice would recover after 24 h. There were no behavioral changes observed in mice in each group before injection and the above symptoms began 7 d after injection. Compared with the normal group, mice in the remaining six groups had low scores in the pole test. The scores between the PD and NC groups did not differ significantly. As compared with the PD group, scores for mice in the miR-96 mimic group were lower while the scores for mice in the miR-96 inhibitor and CACNG5 groups were higher (all *P* < 0.05). There were no significant differences found between the PD group and the miR-96 mimic + CACNG5 groups (*P* > 0.05) (Table 2).

**Table 2:** Behavioral changes at different time points for mice among seven groups

	Number	Before injection	7 d after injection	10 d after injection	12 d after injection	14 d after injection
Normal group	10	8.06 ± 0.71	8.22 ± 0.80	8.17 ± 0.75	8.19 ± 0.86	8.63 ± 0.53
PD group	10	8.46 ± 0.36	6.55 ± 0.63^*^	6.05 ± 0.35^*^	6.51 ± 0.81^*^	7.11 ± 0.68^*^
NC group	10	8.39 ± 0.70	6.56 ± 0.71^*^	6.09 ± 0.49^*^	6.55 ± 0.50^*^	7.06 ± 0.81^*^
miR-96 mimic group	10	8.07 ± 0.37	4.38 ± 0.74^*#^	4.23 ± 0.35^*#^	4.27 ± 0.27^*#^	5.54 ± 0.62^*#^
miR-96 inhibitor group	10	8.11 ± 0.16	3.22 ± 0.71^*#^	3.06 ± 0.44^*#^	3.19 ± 0.32^*#^	4.13 ± 0.59^*#^
CACNG5 group	10	8.25 ± 0.21	3.56 ± 0.17^*#^	3.41 ± 0.46^*#^	3.37 ± 0.18^*#^	4.15 ± 0.45^*#^
miR-96 mimic + CACNG5 group	10	8.30 ± 0.69	6.36 ± 0.66^*^	6.06 ± 0.73^*^	6.60 ± 0.78^*^	7.03 ± 0.67^*^

Note: The test was repeated three times independently, and the one-way ANOVA analysis was used for comparison among multiple groups (*n* = 10)

*PD* Parkinson disease, *NC* Negative control, *miR-96* microRNA-96, *CACNG5* Calcium voltage-gated channel auxiliary subunit gamma 5, *d* Day

^*^P < 0.05 compared with the normal group, ^#^, *P* < 0.05 compared with the PD group

### Inhibition of miR-96 increases substantia nigral cells

**Fig. 3:**
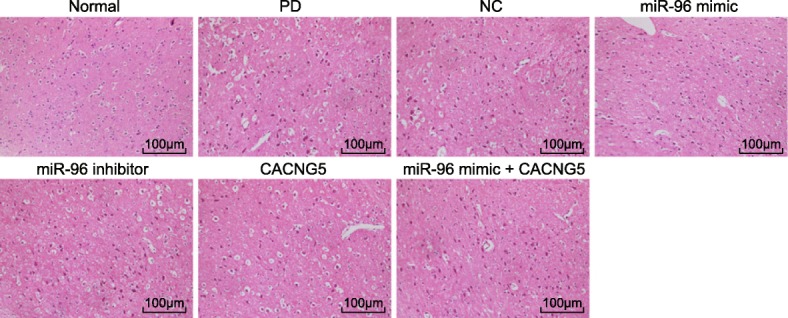
HE staining shows that miR-96 inhibitor increases the number of substantia nigra (× 100). *n* = 10 in each group; HE, hematoxylin-eosin; miR-96, microRNA-96; CACNG5, calcium voltage-gated channel auxiliary subunit gamma 5; PD, Parkinson’ disease; NC, negative control

HE staining was conducted in the brain tissue of the mice in each groups in order to observe the changes of substantia nigral cells. The results showed that the substantia nigra neurons in the normal group were well-distributed and the nucleus had a clear structure. In the PD and NC groups, the substantia nigral cells were significantly decreased and the nucleus of substantia nigral cells presented with pycnosis, hyperchromasia and interstitial edema. In addition, proliferated inflammatory cells, glial cells, and connective tissues were observed in the PD and NC groups. Compared with the PD and NC groups, substantia nigral cells in the miR-96 mimic group had reduced notably, but no obvious reduction was found in the miR-96 inhibitor and CACNG5 groups; while the miR-96 mimic + CACNG5 groups had no significant difference (Fig. 3). The above results indicated that the suppression of miR-96 increases the substantia nigral cells by regulating CACNG5.

### miR-96 inhibition increases TH positive cells in the substantia nigra

**Fig. 4:**
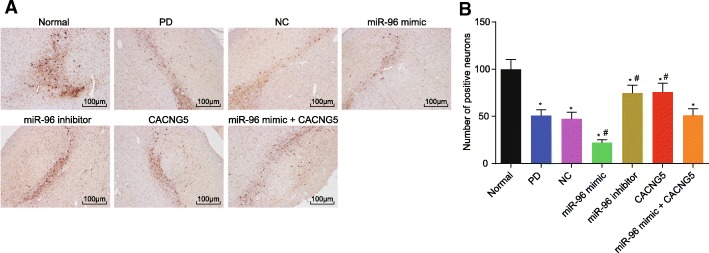
miR-96 inhibitor increases expression of TH in substantia nigra dopaminergic neuronal tissues (× 100). **a** TH immunohistochemistry for dopaminergic neuronal tissues among seven groups; **b** positive TH cells among seven groups under a microscope; ^*^, *P* < 0.05 compared with the normal group, ^#^, *P* < 0.05 compared with the PD and NC groups. The test was repeated three times independently, and the one-way ANOVA analysis was used for comparison among multiple groups (n = 10 in each group). TH, tyrosine hydroxylase; miR-96, microRNA-96; CACNG5, calcium voltage-gated channel auxiliary subunit gamma 5; PD, Parkinson’ disease; NC, negative control

Immunohistochemistry was used to investigate the effect of miR-96 on the number of TH positive cells in PD. Following injection, a number of TH positive cells in nigra could be observed in mice in the normal group and the cytoplasm presented with brownish-yellow TH positive cells. The remaining six groups presented with a remarkable decrease in TH positive cells compared with the normal group (*P* < 0.05). In comparison with the PD and NC groups, TH positive cells in the miR-96 mimic group were significantly reduced (*P* < 0.05), the miR-96 inhibitor and CACNG5 groups had increased TH positive cells (*P* < 0.05), but no evident difference was observed in the miR-96 mimic + CACNG5 group (*P* > 0.05) (Fig. 4). Therefore, the suppression of miR-96 could potentially increase TH positive cells in substantia nigra in PD.

### miR-96 inhibition decreases iNOS positive cell and iNOS expression

**Fig. 5:**
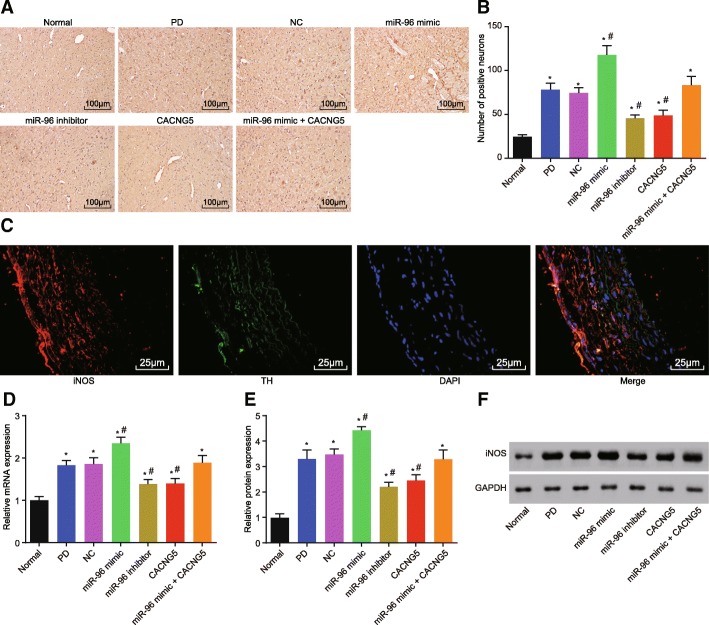
miR-96 inhibitor results in a decrease in the expression of iNOS in dopaminergic neuronal tissues. **a** iNOS immunohistochemistry for dopaminergic neuronal tissues among seven groups; **b** iNOS positive cells among seven groups under a microscope; **c** immunohistochemistry of colocalization analysis between the TH and iNOS; **d** mRNA expression of iNOS among seven groups evaluated by RT-qPCR; **e** protein expression of iNOS among seven groups; **f** protein bands of iNOS among seven groups evaluated by Western blot analysis; ^*^, *P* < 0.05 compared with the normal group, ^#^, *P* < 0.05 compared with the PD and NC groups; Bar = 50 μm. The test was repeated three times independently, and the one-way ANOVA analysis was used for comparison among multiple groups (n = 10 in each group). iNOS, inducible nitric oxide synthase; RT-qPCR, reverse transcription quantitative polymerase chain reaction; miR-96, microRNA-96; CACNG5, calcium voltage-gated channel auxiliary subunit gamma 5; PD, Parkinson’ disease; NC, negative control

iNOS is the response molecule of downstream MAPK. Immunohistochemistry was used to investigate the effect of miR-96 on the iNOS expression. After injection, the normal group presented with a few iNOS positive cells in the substantia nigra. The brownish-yellow iNOS positive cells were located in the cytoplasm. The remaining six groups had more iNOS positive cells than the normal group (*P* < 0.05). Compared with the PD and NC groups, there was a significant decrease in iNOS positive cells in the miR-96 mimic group (*P* < 0.05), while iNOS positive cells in the miR-96 inhibitor and CACNG5 groups were notably decreased (*P* < 0.05); there was no obvious difference observed in the miR-96 mimic + CACNG5 group (*P* > 0.05) (Fig. 5a and b). The aforementioned results suggested that there was co-localization between TH and iNOS (Fig. 5c).

The results obtained from RT-qPCR and Western blot revealed that the remaining six groups had elevated mRNA and protein expression of iNOS when compared with the normal group (*P* < 0.05). As compared with the PD and NC groups, the miR-96 mimic group had increased mRNA and protein expression of iNOS (*P* < 0.05), the miR-96 inhibitor and CACNG5 groups had decreased mRNA and protein expression of iNOS (*P* < 0.05), while the mRNA and protein expression of iNOS had an insignificant alteration in the miR-96 mimic + CACNG5 group (*P* > 0.05) (Fig. 5d, e and f).

### Suppressed miR-96 inhibits the apoptosis of hippocampal neuron

**Fig. 6:**
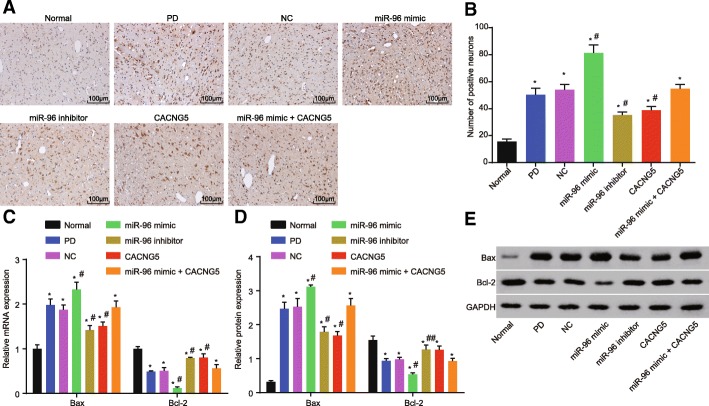
Lower Bax and higher Bcl-2 were found after transfection of miR-96 inhibitor. **a** TUNEL staining of dopaminergic neuronal tissues among seven groups (× 100); **b** positive cells among seven groups under a microscope; **c** mRNA expression of Bax and Bcl-2 among seven groups evaluated by RT-qPCR; **d** protein expression of Bax and Bcl-2 among seven groups evaluated by Western blot; **e** protein bands of Bax and Bcl-2 among seven groups; Bar = 50 μm. The test was repeated three times independently, and the one-way ANOVA analysis was used for comparison among multiple groups (*n* = 10 in each group). ^*^, *P* < 0.05 compared with the normal group, ^#^, *P* < 0.05 compared with the PD and NC groups; TUNEL, terminal deoxynucleotidyl transferase-mediated dUTP nick-end-labeling; RT-qPCR, reverse transcription quantitative polymerase chain reaction; Bcl-2, B-cell lymphoma-2; Bax, Bcl-2 associated protein X; miR-96, microRNA-96; CACNG5, calcium voltage-gated channel auxiliary subunit gamma 5; PD, Parkinson’ disease; NC, negative control

TUNEL staining was employed to detect the effect of miR-96 on the apoptosis of hippocampal neuron. Based on the results, there were a number of brownish yellow apoptotic cells observed in all six groups with the exception of the normal group. The staining was found in the nucleus with a discoloration present in the cytoplasm. There was also occasional nuclear pyknosis, narrowing in nucleus volume, and vacuoles observed in these groups. The normal group only presented with several apoptotic cells. Compared with the PD group, apoptotic cells in the miR-96 mimic group were notably increased (*P* < 0.05), apoptotic cells in the miR-96 inhibitor and CACNG5 groups were reduced (*P* < 0.05), and there was no difference found in the miR-96 mimic + CACNG5 group (*P* > 0.05) (Fig. 6a and b).

The results of RT-qPCR and Western blot showed that as compared with the normal group, mRNA and protein expressions of Bax were elevated but expression of Bcl-2 was decreased in the remaining six groups (all *P* < 0.05). Compared with the PD and NC groups, the miR-96 mimic group had elevated Bax level and decreased Bcl-2 level (all *P* < 0.05), the miR-96 inhibitor and CACNG5 groups had decreased Bcl-2 level but elevated Bax level (all *P* < 0.05), whereas the miR-96 mimic + CACNG5 group presented with no significant difference (all *P* > 0.05) (Fig. 6c, d and e). These results suggested that the inhibition of miR-96 expression could inhibit cell apoptosis of hippocampal neuron via CACNG5 regulation.

### miR-96 inhibition decreases expression of miR-96, p-p38MAPK, p38MAPK and c-Fos while enhancing CACNG5 expression

**Fig. 7:**
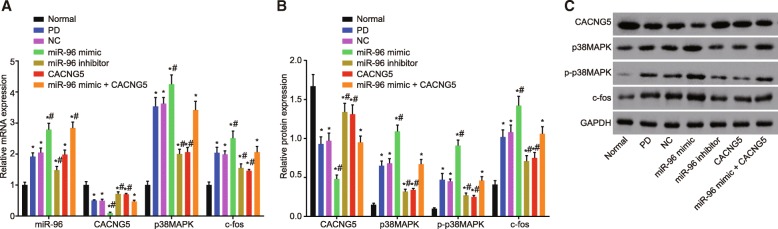
Down-regulation of miR-96 leads to the inhibition of the MAPK signaling pathway through the up-regulation of CACNG5. **a** miR-96 expression and mRNA expression of CACNG5, p38MAPK and c-Fos evaluated by RT-qPCR among seven groups; **b** protein expression of CACNG5, p38MAPK, c-Fos, and p-p38MAPK evaluated by Western blot analysis among seven groups; **c** protein bands of CACNG5, p38MAPK, c-Fos, and p-p38MAPK; GAPDH used as a internal reference. The test was repeated three times independently, and the one-way ANOVA analysis was used for comparison among multiple groups (*n* = 10 in each group). ^*^, *P* < 0.05 compared with the normal group, ^#^, *P* < 0.05 compared with the PD and NC groups; miR-96, microRNA-96; CACNG5, calcium voltage-gated channel auxiliary subunit gamma 5; p38MAPK, p38 mitogen-activated protein kinase; RT-qPCR, reverse transcription quantitative polymerase chain reaction; PD, Parkinson’ disease; NC, negative control

RT-qPCR and Western blot analysis were conducted in order to examine the effect of miR-96 on downstream molecules in the MAPK signaling pathway and determine the influence the activation of the MAPK signaling pathway had on the expression of multiple molecules in its downstream signaling pathway. The results indicate that there was an elevation in the expression of miR-96, p-p38MAPK, p38MAPK and c-Fos, while CACNG5 mRNA and protein expression was reduced in the remaining six groups in comparison with the normal group (all *P* < 0.05). In comparison with the PD and NC groups, the miR-96 mimic group presented with increased levels in the expression of miR-96 and p-p38MAPK, and mRNA and protein expression of p38MAPK and c-Fos, while CACNG5 mRNA and protein expression was reduced (all *P* < 0.05); the miR-96 inhibitor group had elevated CACNG5 mRNA and protein expression but reduced expression of miR-96 and p-p38MAPK, and mRNA and protein expression of p38MAPK and c-Fos (all *P* < 0.05); the CACNG5 group had increased CACNG5 mRNA and protein expression, decreased expression of p-p38MAPK, and mRNA and protein expression of p38MAPK and c-Fos (all *P* < 0.05); there were no significant differences observed in miR-96 expression (*P* > 0.05). The miR-96 mimic + CACNG5 group had elevated miR-96 expression but no evident differences were observed in expression of CACNG5, p-p38MAPK, p38MAPK and c-Fos (all *P* > 0.05) (Fig. 7). These findings highly indicated that the suppression in the expression of miR-96 could inhibit the activation of the MAPK signaling pathway through CACNG5.

## Discussion

PD is a neurodegenerative disease that is commonly accompanied by the loss of dopaminergic neurons in the substantia nigra pars compacta, resulting in characteristic symptoms that are present in PD patients (Son et al. 2012). Alterations in miRNA expression have been reported to contribute to cell cycle control and proteasome activity, leading up to neuronal death (Meza-Sosa et al. 2012). Therefore, the present study was conducted to explore the effects of miR-96 on iNOS and midbrain dopaminergic neurons in mice model of PD. Our findings evidently demonstrated that miR-96 plays a functional role by promoting the activation of iNOS and dopaminergic neuronal apoptosis by means of CACNG5 down-regulation through the MAPK signaling pathway.

Our study found that there was an overexpression of miR-96 in PD mice. The mice miR-96-mimic group also presented with increased expression of p38MAPK, p-p38MAPK and c-fos and reduced CACNG5 expression. Ubhi et al. found that miR-96 is up-regulated in Multiple System Atrophy, a progressive neurodegenerative disorder (Ubhi et al. 2014) and this finding was consistent with our result. It has previously been reported that MAPK signaling pathway is driven by the stimulation of glutamate receptors and that p38MAPK is involved in dopaminergic neuronal death induced by glutamate (Izumi et al. 2009). Lien et al. suggested that glucocorticoids have the ability to significantly reduce the expression of pro-inflammatory cytokines and that there are certain miRNAs including miR-96 that can inhibit the expression of glucocorticoid receptors (Dejager et al. 2014). Roux et al. revealed that p38MAPK is activated by a number of inflammatory extracellular mediators, including cytokines, and chemokines (Roux and Blenis 2004). A key finding indicated that neuroinflammation is a major contributor in the pathogenesis of PD (Qian et al. 2010). c-Fos is an immediate-early gene which regulates various cellular processes including cell proliferation, differentiation and death and the induction of c-Fos expression is partially dependent on p38 and extracellular stimuli-responsive kinase MAPKs (Ely et al. 2011). In addition, CACNG5 was found to be a target gene of miR-96 by a biological website (http://www.targetscan.org) which was further confirmed by means of dual luciferase reporter gene assay.

The results from our study also showed that there was an increase in iNOS positive cells iNOS expression in the miR-96 mimic group while there was a decrease in TH positive cells as compared with the PD group and the NC group. As a rate-limiting enzyme in the dopamine synthesis, TH plays a key role in the central dopaminergic system, as it is able to control voluntary movements and reward-related behaviors (Tokuoka et al. 2011). It has been reported that the reduction of TH activity is related to several neurodegenerative and neuropsychiatric diseases such as Alzheimer’s disease, Doparesponsive dystonia and PD (Skjevik et al. 2014). Yuan et al. indicated that the activation of intracellular signaling proteins like p38MAPK can stimulate the glucose-induced iNOS expression (Yuan et al. 2009). Brito et al. described that nitric oxide, a product of iNOS, regulates glutamate toxicity in primary cortical cultures and contributes to mitochondrial dysfunction, consequently leading to neurodegeneration (Brito et al. 2010). Previous studies suggested that TH gene transcription and expression can be stimulated by glucocorticoid which is inhibited by miR-96 (Dejager et al. 2014; Sheela Rani et al. 2013).

Moreover, the miR-96 mimic group presented with elevated levels in Bax and lowered level of Bcl-2, while it was on the contrary in the miR-96 inhibitor and CACNG5 groups in comparison with the PD and NC groups. In addition, there was a remarkable increase in the apoptotic dopaminergic neurons in the miR-96 mimic group and a notable decrease in the miR-96 inhibitor and CACNG5 groups. As key regulators of mitochondria-mediated apoptosis pathway, Bcl-2 family includes pro-apoptotic and anti-apoptotic members (Chen et al. 2015). Bax and Bcl-2, two pivotal members in the Bcl-2 family, play a functional role as pro-apoptotic protein and anti-apoptotic protein respectively (Zeren et al. 2014). Yang et al. demonstrated that Bcl-2 is a target gene of mmu-miR-96 and that the up-regulation of mmu-miR-96 results in the inhibition of Bcl-2 expression (Yang et al. 2017). Wu et al. reported that p38MAPK was involved in the nuclear translocation of p53 which contributes to Bax induction, leading to rotenone’s neurotoxicity in a PD model (Wu et al. 2013). A previous study provided evidence that the overexpression of miR-96 induces the apoptosis of retinal ganglion cells (Wang and Li 2014).

## Conclusions and perspectives

In conclusion, our study evidently demonstrated that miR-96 activates iNOS and contributes the apoptosis of dopaminergic neuronal by activating the MAPK signaling pathway in a PD mouse model. These findings provide a new theoretical basis in the treatment of PD and alleviating the symptoms associated with it. However, further studies are required in order to confirm our findings regarding the interactions between miR-96 and CACNG5.

## Abbreviations

Bax: Bcl-2 associated protein X
CACNG5: Calcium voltage-gated channel auxiliary subunit gamma 5
DAB: Diaminobenzidine
GAPDH: Glyceraldehyde-3-phosphate dehydrogenase
HE: Hematoxylin-eosin
iNOS: Inducible nitric oxide synthase
MAPK: Mitogen-activated protein kinases
miR-96: MicroRNA-96
miRNAs: MicroRNAs
MPTP: 1-methyl-4-phenyl-1, 2, 3, 6-tetrahydropyridine
Mut: Mutant
NC: Negative control
PBS: Phosphate buffer saline
PBST: Phosphate-buffered saline with Tween-20
PD: Parkinson’s disease
RT-qPCR: Reverse transcription quantitative polymerase chain reaction
SDS-PAGE: Sodium dodecyl sulfate polyacrylamide gel electrophoresis
TUNEL: Terminal deoxynucleotidyl transferase-mediated dUTP nick end labeling
wt: Wild type
